# Physico-chemical characterization of formulations containing endomorphin-2 derivatives

**DOI:** 10.1007/s00726-017-2470-x

**Published:** 2017-07-27

**Authors:** Anna Olejnik, Alicja Kapuscinska, Grzegorz Schroeder, Izabela Nowak

**Affiliations:** Faculty of Chemistry, Adam Mickiewicz University in Poznan, Umultowska 89b, 61-614 Poznań, Poland

**Keywords:** Endomorphin-2, Tetrapeptides, YPFF, Formulations, Release studies

## Abstract

In this study semisolid formulations containing AcYPFF (*N*-acetyl-Tyr-Pro-Phe-Phe-NH_2_) tetrapeptide were obtained and characterized in terms of rheology, stability by multiple light scattering and particle size distribution by laser diffraction. Additionally, the release studies of tetrapeptide from formulations obtained were performed. The influence of different factors such as semisolid and membrane type on tetrapeptide release rate was examined. The release experiments of tetrapeptide modified with palmitoyl group (PalmYPFF) were also carried out. The results proved that formulation type and its rheological properties strongly determined the permeation process of the tetrapeptide. The fastest release of tetrapeptide was observed from hydrogel that had the lowest viscosity. The kinetic data of tetrapeptide released from oil-in-water (o/w) and water-in-oil (w/o) emulsions prepared at elevated temperature showed good fit to the Higuchi equation, whereas when AcYPFF was released from oil-in-water (o/w) emulsion prepared with the addition of auto-emulsifier high linearity with Korsmeyer–Peppas model was observed. While when tetrapeptide was released from Hydrogel the most suitable model was the first-order kinetics. It was suggested that mechanism that led to the release of tetrapeptide from all formulations was non-Fickian diffusion transport. The presence of palmitoyl group changed the solubility of tetrapeptide both in formulation and receptor fluid and thus the release rate of active compound was modified.

## Introduction

In recent years, the low molecular weight peptides have revolutionized the skincare and pharmaceutical industries and have become significant ingredients applied in topical formulations (Lupo and Cole 2007). The great demand for these compounds is connected with their activity because peptides are involved in many natural processes with relevance to skincare (Zhang and Falla 2009; Fields et al. 2009). Low molecular weight peptides applied in topical applications can be divided into three main groups depending on their activity—signal peptides, carrier peptides and neurotransmitter peptides (Olejnik et al. 2013; Lupo 2005). Acetyl-Tyr-Pro-Phe-Phe-NH_2_ (AcYPFF) is a synthetic derivative of endomorphin-2 (H-Tyr-Pro-Phe-Phe-NH_2_) that is known as opioid peptide (Van Dorpe et al. 2010), which is applied as a pharmaceutical agent. Endomorphin-2 is a naturally occurring amidated tetrapeptide (Zadina et al. 1997). In general, endomorphins exhibit high affinity for the µ-opioid receptor (Fichna et al. 2008); therefore, they are responsible for various pharmacological effects including analgesia. However, the cosmetic application of the oligopeptides such as R1-Tyr-Pro-Phe-Phe-NH_2_ and their derivatives were reported for the first time in the patent no. WO 2007051550 A1 (Gillon et al. 2007). The most preferred structure of this invention was the oligopeptide *N*-acetyl-Tyr-Pro-Phe-Phe-NH_2_. The cosmetic emulsion containing AcYPFF were obtained to be applied on sensitive skin and to ameliorate existing symptoms of sensitive skin. AcYPFF was also applied as the hair treatment agent which is added to the composition in the form of commercial products: Skinasensyl^®^ PW LS 9852 or LS 9747 (Laboratoires Serobiologiques) (Kleen 2010). An acetylated tetrapeptide (Skinasensyl^®^) was synthesized to decrease the stimulation of the skin’s nerve endings and to relieve sensitive skin and thus to enhance its tolerance (Gilles et al. 2009). Hypersensitive skin is more responsive to external factors such temperature variations, sun or UV radiations, various drugs and cosmetic products. This type of skin can react on these factors with subjective symptoms such as itching, burning, tingling or pain sensations. It is thought that neurogenic hyperreactivity could be related to the release of neuropeptides—calcitonin gene-related peptide (CGRP) (Boulais and Misery 2008). It was demonstrated in in vitro studies that AcYPFF could decrease the release of calcitonin gene-related peptide from sensory neurons (Gilles et al. 2009). The effectiveness of AcYPFF as soothing agent was also proved in in vivo studies. For this purpose cutaneous application of capsaicin (main capsaicinoid in chili peppers that has irritating properties causing redness, itching, burning and other uncomfortable skin feeling) was performed on volunteers. Volunteers presenting hypersensitivity to capsaicin have applied 0.0015 wt% Skinasensyl on the nasolabial fold twice a day. After 4 days the sensitivity threshold was determined by applying different, increasing concentration of capsaicin in solution. As a result, skin hypersensitivity decrease was observed on the side of face, where AcYPFF was applied. The results proved that tetrapeptide could be applied to relieve sensitive skin by decreasing its hyperreactivity to external factors (Gilles et al. 2009).

AcYPFF exhibits interesting properties as an active ingredient of topical formulations. However, to the best of our knowledge there has been no research concerning the stability of these formulations and in vitro release test of the active compound from these topical semisolids. Therefore, the aim of this study was to develop and characterize the novel semisolid formulations for tetrapeptide AcYPFF (Table 1) and to carry out the release studies of the active compound. These experiments are useful in the design and development of new preparation and also in quality control (Olejnik et al. 2012). The influence of different factors such as semisolid and membrane type on tetrapeptide release rate was examined. Additionally, the permeation ability of tetrapeptide modified with palmitoyl group was also studied (Table 1).

**Table 1:**
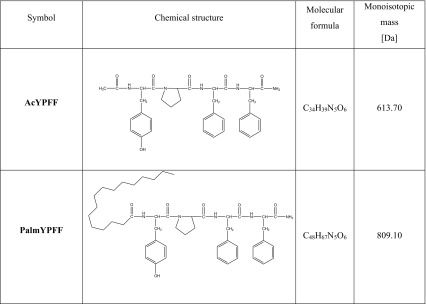
Chemical structure of tetrapeptides

It should be mentioned that typical emulsion consists of two phases (aqueous and oil phases), one of which is dispersed throughout the second phase. If the oil droplets are dispersed throughout the water phase the emulsion is called oil-in-water (o/w). If the water is dispersed in oil continuous phase the system is termed water-in-oil emulsion (w/o) (Khan et al. 2011). Emulsions are thermodynamically unstable; therefore, the third agent known as emulsifier is added to stabilize this system (Agarwal and Rajesh 2007). O/W emulsions are non-greasy and are mostly applied to give the cooling effect. On the other hand, w/o emulsions provide an occlusive effect and hydrate the stratum corneum; therefore, they are applied for the treatment of dry skin. Water-soluble active compounds are more quickly released from o/w emulsions. However, w/o emulsions are better vehicles for release of oil-soluble drugs (Khan et al. 2011).

## Materials and methods

### Materials

The tetrapeptide AcYPFF and PalmYPFF were synthesized by Lipopharm (Poland). Potassium phosphate buffer was purchased from J. T. Baker^®^ (USA). Synthetic membranes made of nitrocellulose (Protran BA 85) and polytetrafluoroethylene (PTFE) were purchased from Whatman (USA). Nylon membrane was obtained from GVS Filter Technology (USA). Cuprophan (regenerated cellulose) and polyvinylidene fluoride (PVDF) were purchased from Agilent Technologies (USA) and GemaMedical (Israel), respectively.

### ESI mass spectrometry

The ESI mass spectrometric measurements of the endomorphin-2 derivatives were performed by the Waters/Micromass (Manchester, UK) ZQ equipped with a Harvard Apparatus syringe pump. Nitrogen was employed as the nebulizing and desolvation gas, whereas argon with high purity was employed as a collision gas. The temperatures of the electrospray source and desolvation gas were 100 and 200 °C, respectively. The capillary voltage was 3500 V and pump flow was 5 µL min^−1^. ESI mass spectra were acquired in both positive and negative modes.

### Semisolid preparation

Semisolids were prepared according to the procedure presented in the previous paper (Olejnik et al. 2015a). Chemical compositions of the formulation are summarized in Table 2.

**Table 2:** Chemical compositions of the formulations: oil-in-water (o/w) emulsions, water-in-oil (w/o) emulsion and hydrogels

Sample name	Composition	Commercial name	Company	Quantity (%, ±0.01)
Emulsion 1 AcYPFF (o/w)	Polyglyceryl-3 methylglucose distearate	Tego Care 450	Evonik	3.25
	Cetearyl alcohol	Tego Alkanol 1618	Evonik	2.50
	Glyceryl stearate	Tegin 4100 Pellets	Evonik	2.50
	Ethylhexyl stearate	Cetiol 868	Cognis	10.00
	Squalane	Fitoderm	Cognis	5.25
	Glycerin		Chempur	5.50
	Distilled water			70.00
	Citric acid		Sigma-Aldrich	0.50
	AcYPFF		Lipopharm	0.50
Emulsion 2 AcYPFF (w/o)	Cetearyl alcohol	Tego Alkanol 1618	Evonik,	10.00
	Paraffin oil		Sigma-Aldrich	15.00
	Isopropyl palmitate		Sigma-Aldrich	5.00
	Vaseline		Sigma-Aldrich	49.00
	Distilled water			20.00
	Citric acid		Sigma-Aldrich	0.50
	AcYPFF		Lipopharm	0.50
Emulsion 3 AcYPFF (o/w with auto-emulsifier)	Sodium acrylate/sodium acryloyldimethyl taurate copolymer & isononyl isononanoate	Creagel EZ IN	Créations Couleurs (CC)	10.00
	Hydrogenated polydecene	Alphaflow 20	CC	17.00
	Distilled water			72.00
	Citric acid		Sigma-Aldrich	0.50
	AcYPFF		Lipopharm	0.50
Hydrogel AcYPFF	Carbomer (crosslinked polyacrylate polymer)	Tego Carbomer 340 FD	Evonik,	0.50
	Sodium hydroxide (10% solution)		Chempur	1.00
	Isopropanol		Sigma-Aldrich	25.00
	Distilled water			73.00
	AcYPFF		Lipopharm	0.50
Hydrogel PalmYPFF	Carbomer (crosslinked polyacrylate polymer)	Tego Carbomer 340 FD	Evonik,	0.50
	Sodium hydroxide (10% solution)		Chempur	1.00
	Isopropanol		Sigma-Aldrich	25.00
	Distilled water			73.00
	PalmYPFF		Lipopharm	0.50

### Characterization of obtained formulations

#### Determination of pH

The pH values of semisolid formulations were determined by a pH meter (Testo, Australia). The measurements were conducted at room temperature (RT) in triplicate and the average value was determined.

#### Viscosity

The viscosity measurements were performed in triplicate at room temperature using a rotational viscometer equipped with a temperature sensor (RC02 Viscometer, Rheotec, Germany).

### Stability test by multiple light scattering

The stability studies of emulsions obtained were performed directly after preparation of the emulsions and at different times for 60 days using Turbiscan Lab Expert (Formulaction, France). Multiple light scattering was applied to measure the stability of semisolid formulations at room temperature (RT). Turbiscan Stability Index (TSI) was used to compare the general behaviour of samples and it was calculated as the sum of all of the destabilization processes occurring in the emulsion studied (Zhao et al. 2014; Carbone et al. 2015).

### Particle size distribution analysis by laser diffraction

The particle size distributions of formulations containing tetrapeptide AcYPFF were determined using Mastersizer 2000 (Malvern, UK) equipped with a hydrodispersion unit. The measurements were performed in triplicate at room temperature in distilled water. The mean droplet diameter was presented as *d*_3,2_ known as the Sauter diameter which gives information about an average of particle size of sample studied (Perex-Mosqueda et al. 2015; Olejnik et al. 2015b).

### Release studies

Release tests were performed with USP Apparatus 2 (Agilent Technologies DS 708) connected with UV–Vis Cary 50 Bio (Varian, USA). The appropriate semisolid formulation containing AcYPFF was placed into the enhancer cell and covered with the selected synthetic membrane. Five kinds of synthetic membranes were used in these studies, based on cellulose such as Cuprophan, nitrocellulose and based on other polymers such as nylon, PTFE and PVDF. The membranes were soaked in receptor medium for 1 h before use. As the medium a mixture of phosphate buffer (at pH 5.8) and ethanol in the ratio of 65:35 was used. The medium was maintained at 32.0 °C ± 0.5 °C and stirred at 100 rpm. The concentration of released AcYPFF or PalmYPFF tetrapeptides was spectrophotometrically monitored at 279 nm. The absorbance of the sample aliquots was used to assess the amount of compound released at each time point.

### Kinetic calculations

The release data were fitted with various kinetic models such as zero-order (% of AcYPFF release vs. time), first-order (log of % AcYPFF remaining vs. time), Higuchi’s model (% of AcYPFF release vs. square root of time), Korsmeyer–Peppas model (log of % AcYPFF release vs. log time). For each model *R* ^2^ values were determined. In the Korsmeyer–Peppas model, the *n* value was applied to characterize the tetrapeptide release mechanism as described below:

- *n* < 0.5 (0.45)—quasi-Fickian diffusion,
- *n* = 0.5 (0.45)—diffusion mechanism,
- 0.5 (0.45) < *n* < 1—non-Fickian diffusion,
- *n* = 1 (0.89)—case II transport (zero-order release),
- *n* > 1 (0.89)—super case II transport (Sahoo et al. 2012; Dash et al. 2010; Goscianska et al. 2016).

Additionally, for the tetrapeptide release through different membranes the rate constant (*k*) and the peptide half-time release were calculated from kinetic curves by the Guggenheim’s method (Schwetclick 1971; Guggenheim 1926).

## Results and discussion

### Characterization of endomorphin-2 derivatives

**Fig. 1:**
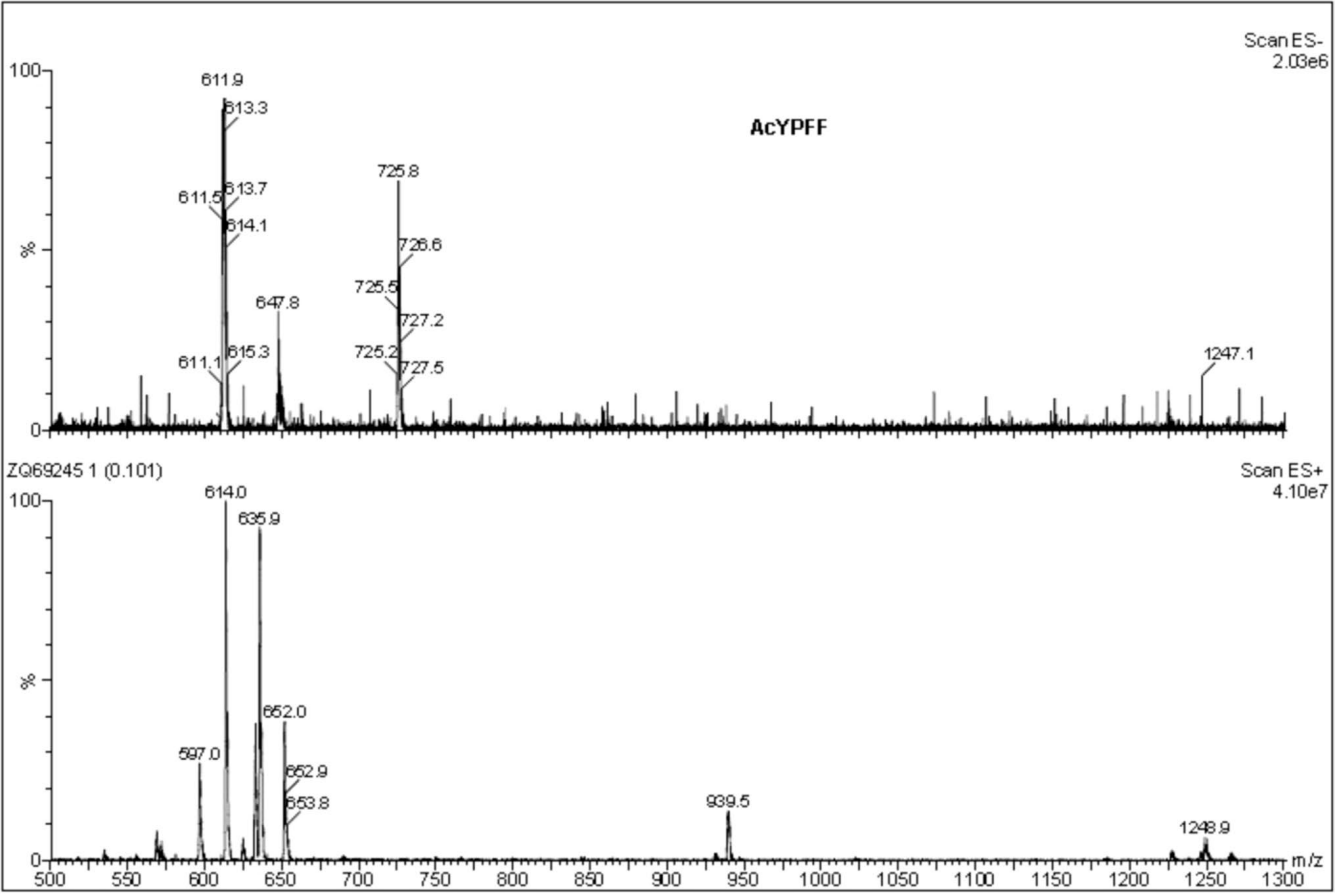
ESI mass spectra of AcYPFF ([M + H]^+^ 614.0, [M + Na]^+^ 635.9, [M + K]^+^ 652.0, [2 M + Na]^+^ 1248.9 and [M-H]^−^ 611.9, [M-H + HCl]^−^ 647.8, [M-H + CF_3_COOH]^−^ 725.8)

The active compounds were characterized by ESI mass spectrometry. The mass spectra of AcYPFF (MW = 613) are presented (Fig. 1). The positive ion mode of AcYPFF shows a protonated molecular ion [M + H]^+^ at *m*/*z* = 614.0 and a molecular ion associated with sodium [M + Na]^+^ at *m*/*z* = 635.9. An additional signal at *m*/*z* = 652.0 corresponds to the potassium adduct [M + K]^+^ and signal at *m*/*z* = 1248.9 corresponds to dimer of AcYPFF associated with sodium [2 M + Na]^+^. In the case of negative ion mode, a signal at *m*/*z* 611.9 corresponds to deprotonated molecular ion [M–H]^−^. The signal at *m*/*z* 647.8 represents the hydrochloride attachment ion [M–H + HCl]^−^ and the signal at *m*/*z* 725.8 corresponds to [M–H + CF_3_COOH]^−^.

**Fig. 2:**
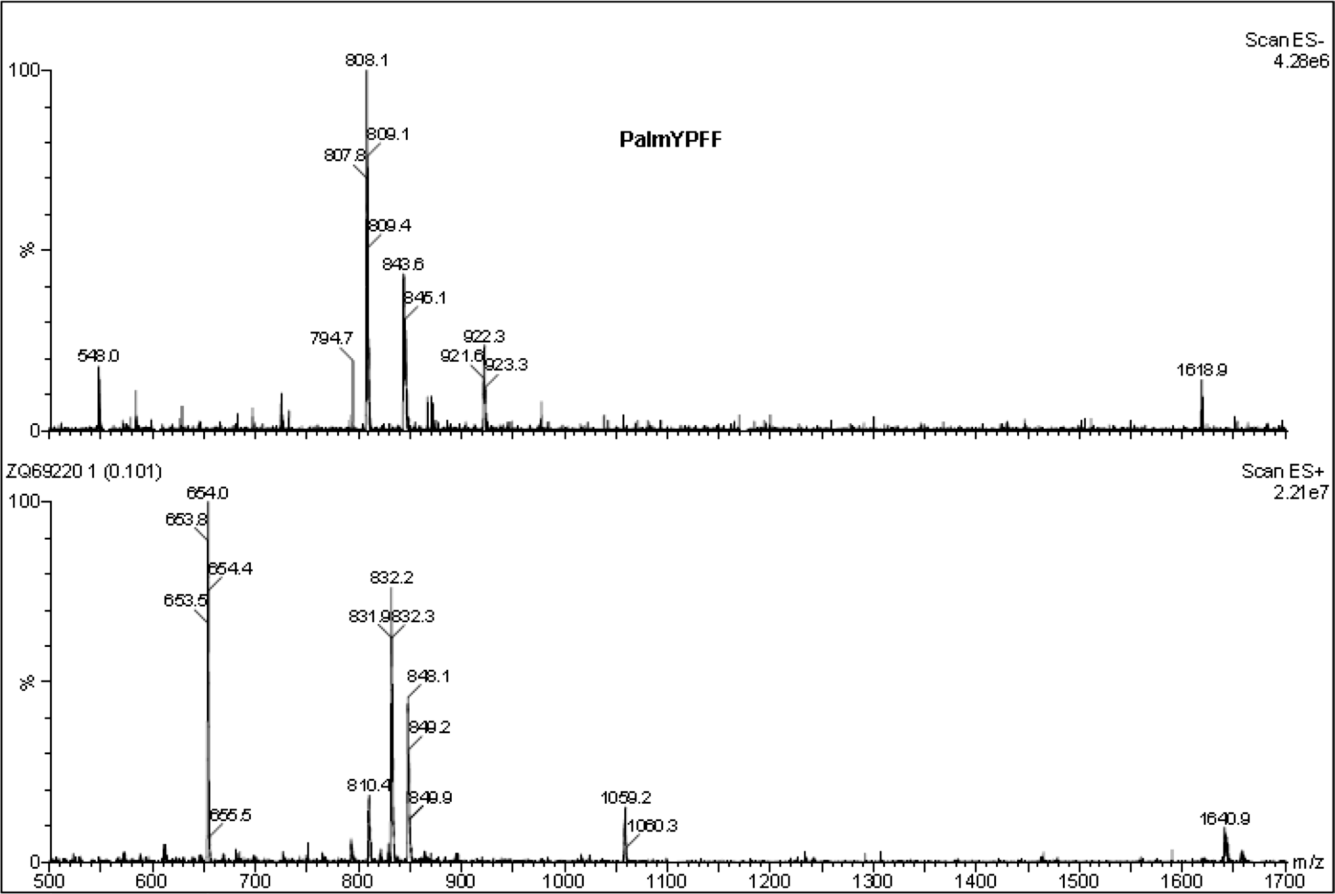
ESI mass spectra of PalmYPFF ([M + H]^+^ 810.4.0, [M + Na]^+^ 832.2, [M + K]^+^ 848.1, [2 M + Na]^+^ 1640.9 and [M-H]^−^ 808.1, [M-H + HCl]^−^ 843.6, [M-H + CF_3_COOH]^−^ 922.3)

The ESI mass spectra of PalmYPFF (MW = 809) were recorded (Fig. 2). The positive ion mode presents a protonated molecular ion [M + H]^+^ at *m*/*z* = 810.4 and a molecular ion associated with sodium [M + Na]^+^ at *m*/*z* = 832.2. An additional, signal at *m*/*z* = 848.1 corresponds to the potassium adduct [M + K]^+^ and signal at *m*/*z* = 1640.9 represents dimer of PalmYPFF associated with sodium [2 M + Na]^+^. In the negative ion mode a signal at *m*/*z* 808.1 corresponds to deprotonated molecular ion [M–H]^−^. Additionally at *m*/*z* = 843.6 [M–H + HCl]^−^ was detected and at *m*/*z* 922.3 [M–H + CF_3_COOH]^−^ was identified.

### Physicochemical characterization

The studies were performed on formulations with and without the active compounds. The results proved that the addition of tetrapeptide to the base of each formulation did not change the physicochemical parameters of semisolids. Additionally, the base of each formulation without the active compound was also prepared. The study was performed on five semisolid formulations such as three emulsions and hydrogel containing AcYPFF tetrapeptide and hydrogel with PalmYPFF tetrapeptide. To compare the release results PalmYPFF was also introduced to Emulsions 1–3. The concentration of tetrapeptide in formulations was 0.5%. First the formulations obtained were subjected to physicochemical analyses. The results of viscosity measurements and pH values are shown in Table 3. Variable skin pH values were reported in the literature, all in the acidic range of pH 4–7 (Lambers et al. 2006). The pH value of semisolid formulation used for skincare application should be between 5 and 6 (Akhtar et al. 2010). The pH values of preparations obtained were around 6.50; therefore, they can be recommended for dermal application. The highest viscosity was observed for Emulsion 2. These results can be explained by its compositions. Sample 2 is a water-in-oil emulsion, which consists mostly of oil phase ingredients such as vaseline, paraffin oil, cetearyl alcohol and isopropyl palmitate. The viscosity of Emulsion 1 was regulated by cetearyl alcohol. In the case of Emulsion 3 Creagel EZ IN (auto-emulsifier) influenced its viscosity. The lowest viscosity was observed for Hydrogel that consists of carbomer (crosslinked polyacrylic acid). It should be mentioned that the addition of AcYPFF did not significantly change the viscosity of the bases.

**Table 3:** pH values, viscosity and diffusion coefficient (*D*) of the obtained semisolid formulations—oil-in-water (o/w) emulsions, water-in-oil (w/o) emulsion and hydrogels

Sample	pH	Viscosity (mPa s)	*D* × 10^−14^ (m^2^ s^−1^)
Emulsion 1 AcYPFF (o/w)	6.50 ± 0.05	41 100 ± 500	10.23
Emulsion 2 AcYPFF (w/o)	6.49 ± 0.04	107 400 ± 1 000	3.91
Emulsion 3 AcYPFF (o/w with auto-emulsifier)	6.51 ± 0.06	25 000 ± 400	16.83
Hydrogel AcYPFF	6.50 ± 0.02	15 500 ± 500	27.14
Hydrogel PalmYPFF	6.50 ± 0.05	15 500 ± 700	24.22

With regard to the viscosity measurements the diffusion coefficient of the tetrapeptides was also calculated. Based on Einstein–Smoluchowski equation (Eq. 1), the diffusion coefficients (*D*) for the diffusion of the tetrapeptide AcYPFF from various semisolid formulations were calculated:

1 $$D = \frac{kT}{6\pi r\eta }\left( {{\text{m}}^{2} {\text{s}}^{ - 1} } \right),$$

where *D* diffusion coefficient (m^2^ s^−1^), *T* temperature (K), *K* Boltzman’s constant, *r* radius of the spherical particle (Å) and *η* viscosity (Pa s).

The tetrapeptide radius was determined by knowing its volume (626.29 Å^3^) and using the equation:

2 $$r = \sqrt[3]{{\frac{3V}{{4\uppi}}}}\left( {\AA} \right),\;{\text{where }}V - {\AA}^{3} .$$

The radius of the tetrapeptide was 5.31 Å. The diffusion coefficients calculated based on Einstein–Smoluchowski equation are shown in Table 3 for both AcYPFF and PalmYPFF. There was a correlation between the D coefficient and viscosity. The higher the viscosity, the smaller is the diffusion coefficient. These results suggest that rheological properties of formulation can strongly influence the release rate of tetrapeptide.

### Stability studies by multiple light scattering

**Fig. 3:**
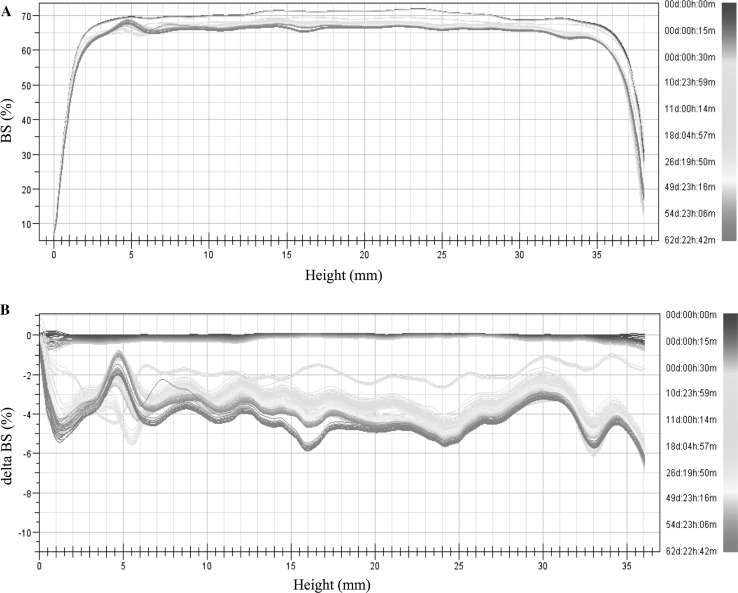
Backscattering (BS) and delta BS profiles of Emulsion 1 measured at room temperature (RT)

**Fig. 4:**
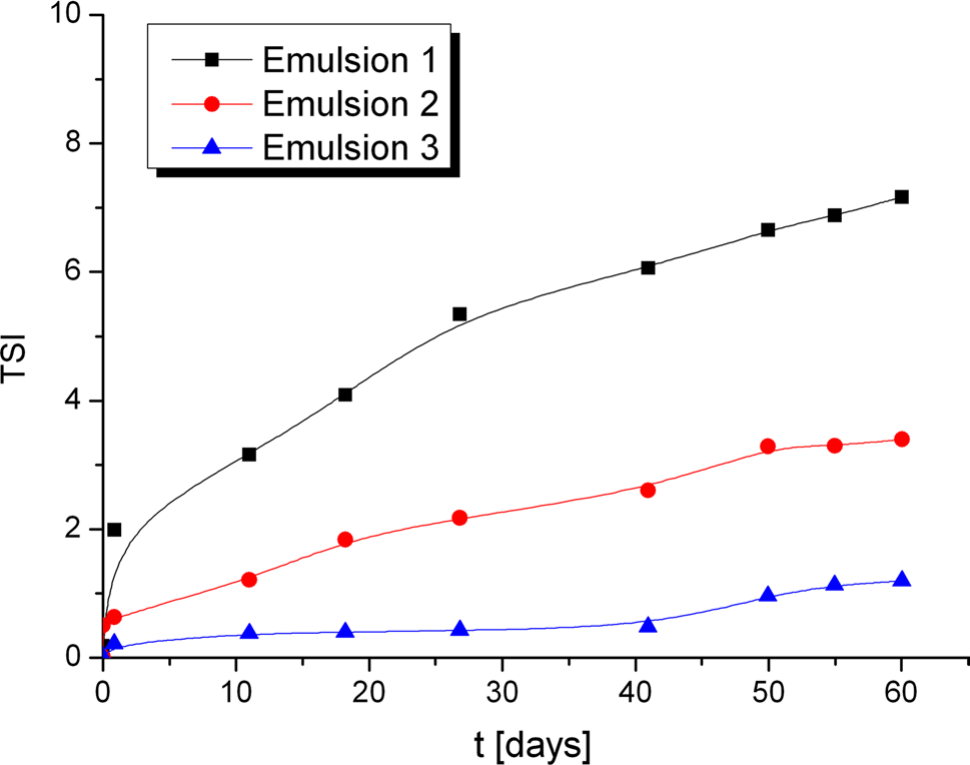
Turbiscan Stability Index of formulation obtained over time

The stability of emulsions obtained was determined by multiple light scattering. Using this method the instability can be detected faster than visually (Santos et al. 2013; Liu et al. 2011). After the measurements the backscattering (BS) curves versus sample height are obtained (non-reference mode). To identify the changes occurring in the sample the obtained data are analysed in the reference mode (delta BS vs. sample height), where the results are compared with the first measurement. In Fig. 3 the BS (backscattering) and delta BS profiles of Emulsion 1 are presented. The first measurement is shown as a blue line and the next measurements are depicted as lines in different colours. Therefore, the changes in stability over time can be determined. In Fig. 3b the flocculation phenomenon can be observed. During 60 days of storage the changes in the backscattering intensity along the whole height of the sample were observed that correspond to the increase of particle size. To compare the stability of different emulsions Turbiscan Stability Index (TSI) can be used, which is calculated on the basis of all changes occurring in the sample. TSI global results of three semisolid formulations are presented in Fig. 4. It should be mentioned that the lower the TSI value, the more stable is the sample. The lowest TSI value was observed for Emulsion 3. This could be explained by the presence of the auto-emulsifier (Creagel EZ IN) that enhances its stability. It should be mentioned that according to Mengual et al. (1999) the sample is treated as unstable when the variation of back scattering is greater than 10%. On the basis of this statement, the emulsions obtained can be considered as stable.

### Particle size distribution by laser diffraction

All the formulations obtained were analysed to determine their particle size distribution. The results are presented in Table 4 in the form of percentages of *d*(0.1), *d*(0.5) and *d*(0.9) that are defined as follows:

**Table 4:** Particle size distribution of formulations containing AcYPFF

Formulation	*d*(0.1) (μm)	*d*(0.5) (μm)	*d*(0.9) (μm)	*D*(3.2) (μm)	*D*(4.3) (μm)
Without sonification					
Emulsion 1	3.59 ± 0.02	11.97 ± 0.11	28.32 ± 0.29	7.82 ± 0.06	14.15 ± 0.12
Emulsion 2	9.16 ± 0.21	38.08 ± 1.21	154.13 ± 20.89	20.54 ± 0.51	65.89 ± 9.63
Emulsion 3	1.67 ± 0.00	3.56 ± 0.00	6.65 ± 0.00	3.00 ± 0.00	3.90 ± 0.00
Hydrogel	26.80 ± 0.31	46.47 ± 0.59	80.42 ± 2.74	42.57 ± 0.33	50.77 ± 0.97
After sonification					
Emulsion 1	0.21 ± 0.01	3.42 ± 0.27	9.26 ± 0.27	0.78 ± 0.07	4.11 ± 0.21
Emulsion 2	0.29 ± 0.01	4.94 ± 0.16	13.749 ± 0.43	1.18 ± 0.05	6.23 ± 0.21
Emulsion 3	1.62 ± 0.01	3.44 ± 0.02	6.460 ± 0.03	2.90 ± 0.01	3.78 ± 0.02

- *d*(0.1) (μm)—10% of the particle distribution is below this value,
- *d*(0.5) (μm)—50% of the distribution above this value and 50% below it),
- *d*(0.9) (μm)—90% of the particle distribution is below this value (Goscianska et al. 2015).

**Fig. 5:**
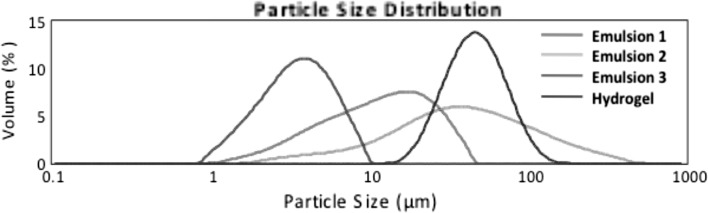
Particle size distribution of formulations obtained measured without ultrasound treatment

**Fig. 6:**
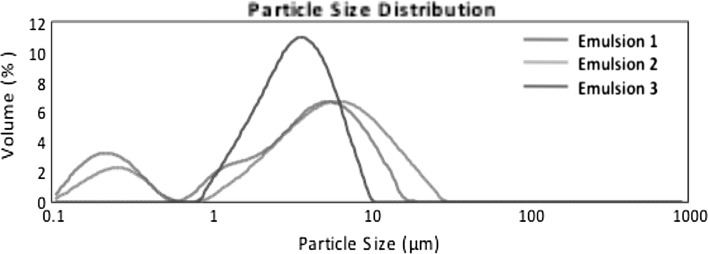
Particle size distribution of formulations obtained measured with usage of ultrasounds 20 kHz

Furthermore, *d*_3,2_ (Sauter diameter) gives information about an average of particle size of sample. The highest value of mean droplet diameter was observed for Hydrogel and the smallest value of the Sauter diameter was detected for Emulsion 3. Figure 5 presents the particle size distribution of formulations obtained. A monomodal particle size distribution pattern was observed for all semisolids. Emulsion 2 revealed a very broad particle size distribution in the range of 5–500 μm whereas Emulsion 3 gave a narrow distribution in the range of 0.9–10 μm. Additionally, the measurements were performed after sonification treatment (ultrasound at 20 kHz) to break up the potential agglomerates. It could be observed in Fig. 6 that in the case of Emulsions 1 and 2 under the influence of ultrasounds the volume fraction of particles of smaller size increased and the particle size distribution pattern changed from monomodal to bimodal. It gives information that in both Emulsions (1 and 2) the large agglomerates were broken down. However, in the case of Emulsion 3 the particle size distributions remained unchanged after sonification. These results are in line with the data obtained by multiple light scattering where almost no changes were observed in particle size after 60 days of storage at RT.

### Release studies of tetrapeptide

#### The influence of formulation type on the release rate of tetrapeptide

**Fig. 7:**
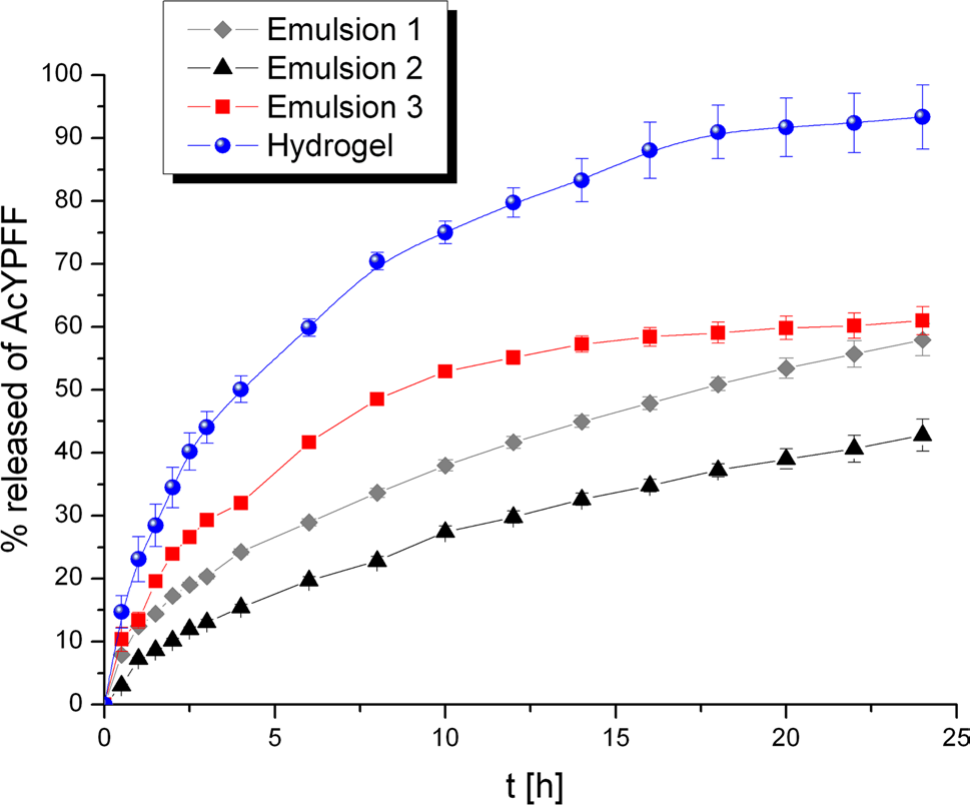
Release profiles of AcYPFF from various formulations (Emulsions 1–3 and Hydrogel) through Cuprophan membrane

The influence of formulation type on release kinetics of tetrapeptide is presented in Fig. 7. It can be observed that tetrapeptide permeated much faster from Hydrogel than from Emulsions. In the case of Hydrogel after 4 h, half of the total mass of AcYPFF was released. Afterwards the percentage of the tetrapeptide in the receptor medium increased to reach around 93% after 24 h. A slower diffusion process of AcYPFF was observed from Emulsion 3. After 9 h half of the total mass of tetrapeptide was released to the receptor medium to finally reach 61%. On the other hand, only 50% of the total mass of AcYPFF was diffused from Emulsion 1 after 18 h. However, the smallest amount of AcYPFF was released from Emulsion 2 that could be explained by its compositions. This formulation consists mostly of the oil phase ingredients (79% w/w); therefore, its viscosity is high that influences the release process of tetrapeptide. Higher concentration of the oil ingredients slowed the release of active compound. The release results showed that the viscosity of formulation strongly influences the release rate of active compound that was also suggested by calculating the diffusion coefficient based on Einstein–Smoluchowski equation. Hydrogel has the diffusion coefficient and the lowest value of D exhibited Emulsion 2 (Table 3). Taking into account these calculations it could be stated that the higher the value of the diffusion coefficient, the faster is the permeation of the tetrapeptide to the medium. Therefore, the Einstein–Smoluchowski equation could be used to predict from which formulation the diffusion process of active compound would be the fastest. These outcomes are in accordance with the data presented in the previous paper where it was indicated that the higher the viscosity of the semisolid, the slower is the permeation of the tetrapeptide through the membrane (Olejnik et al. 2015a).

The kinetic models used to describe the AcYPFF release from various semisolid formulations are shown in Table 5. To investigate the mechanism of tetrapeptide diffusion from different semisolids, the release results were fitted with mathematic equations such as zero-order, first-order, Higuchi’s and Korsmeyer–Peppas model. The “*n*” value obtained from the slope of the plot calculated based on Korsmeyer–Peppas model indicates the release mechanism of the active compound. Taking into account the regression coefficients, the release data were in favour of Higuchi model for Emulsion 1 and Emulsion 2. In the case of Emulsion 3 the highest value of *R* ^2^ was observed for the Korsmeyer–Peppas model. However, for Hydrogel the most suitable model was the first-order kinetics. The values of “*n*” for all semisolid formulations were >0.5 and <1, which indicates the non-Fickian diffusion transport of tetrapeptide. According to Peppas the non-Fickian transport corresponds to a combination of both diffusion and erosion of drug release (Peppas 1985).

**Table 5:** Kinetic models used to describe AcYPFF release from formulations obtained

Formulation	Zero-order kinetics	First-order kinetics	Higuchui model	Korsmeyer–Peppas model		Type of transport
	Regression coefficient (*R* ^2^)				*n*	
Emulsion 1	0.968	0.989	0.999	0.998	0.506	Non-Fickian diffusion
Emulsion 2	0.972	0.987	0.998	0.981	0.638	Non-Fickian diffusion
Emulsion 3	0.890	0.935	0.971	0.980	0.505	Non-Fickian diffusion
Hydrogel	0.915	0.996	0.985	0.988	0.506	Non-Fickian diffusion

#### The influence of membrane type on the release of AcYPFF

**Fig. 8:**
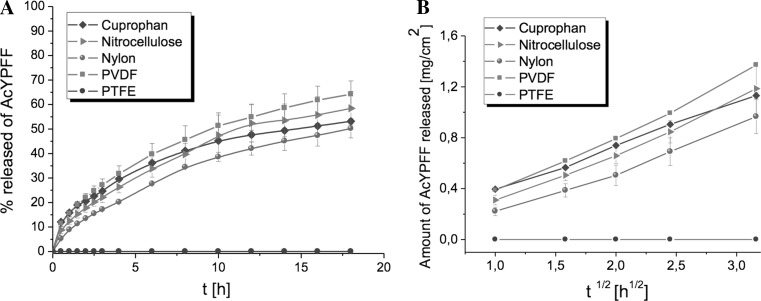
Release profiles of tetrapeptide AcYPFF from Emulsion 1 through five synthetic membranes (Cuprophan, nitrocellulose, nylon, PVDF and PTFE) (**a**) and release kinetics according to the Higuchi’s model (**b**)

The selection of the appropriate membrane is crucial while developing the methodology of release tests. So far different kinds of synthetic membranes were applied in release studies such as cellulose based membranes (Coska et al. 2005; Djordjevis et al. 2005; Arellao et al. 1998; Ng et al. 2010) and membranes based on synthetic polymers (Christensen et al. 2011; Thakker and Chen 2003; Yoshida et al. 2004; Sawant et al. 2010). However, there is no standard membrane that could be applied for the release studies of each active compound. Additionally, on 11 March 2013 the absolute prohibition to use cosmetic substances tested on animals in European Union was introduced (European Commission 2013). Therefore, it is essential to find alternative method to determine the release of active ingredient from formulations and to choose suitable membrane for diffusion studies that will be synthetic and non-animal based. To assess the release profiles of AcYPFF five different synthetic membranes were applied such as Cuprophan, nitrocellulose, nylon, PTFE and PVDF membranes. The Emulsion 1 was selected to perform these experiments. The release profiles of tetrapeptide AcYPFF through synthetic membranes are presented in Fig. 8. In Table 6 the kinetic data are shown. High linearity with Higuchi model was observed when the AcYPFF was released through Cuprophan, nitrocellulose, nylon and PVDF where correlation coefficient was above 0.99. No diffusion of tetrapeptide was observed thorough PTFE membrane due to its hydrophobic nature, high thickness and peculiar structure. The PTFE membrane was analysed in detail by atomic force microscopy in the previous studies (Olejnik and Nowak 2015) where similar results were obtained when AcPPYL was selected for release studies through this membrane. The release rate of tetrapeptide AcYPFF from Emulsion 1 was as follows: PVDF > nitrocellulose > Cuprophan > nylon. The highest amount of AcYPFF was released from PVDF because based on previous studies (Olejnik and Nowak 2015) this membrane had slightly bigger pore size than other membranes. The slowest release rate of AcYPFF was observed through nylon membrane. As it was explained in previous studies, the presence of the additional substructure inside the pores of nylon membrane could be responsible for limited diffusion of the active compound to the receptor fluid.

**Table 6:** Release characteristics of AcYPFF from different types of membranes

Type of membrane	Release rate Higuchi’s model (mg cm^−2^ h^−1^)	Regression coefficient (*R* ^2^)	*k* (h^−1^)^a^	% released after 18 h	*t*_1/2_ (h)
Cuprophan	0.67 ± 0.01	0.998	0.27	53.15 ± 1.05	14.50
Nitrocellulose	0.75 ± 0.06	0.993	0.36	58.43 ± 6.82	10.50
Nylon	0.64 ± 0.53	0.991	0.35	50.23 ± 3.86	18.00
PVDF	0.90 ± 0.01	0.995	0.38	64.22 ± 5.44	9.50
PTFE	nd^b^	nd	nd	nd	nd

^a^ Release constant calculated by the Guggenheim’s method for the initial 4 h

^b^ Not determined

#### The influence of the functional group on the release of tetrapeptide

**Fig. 9:**
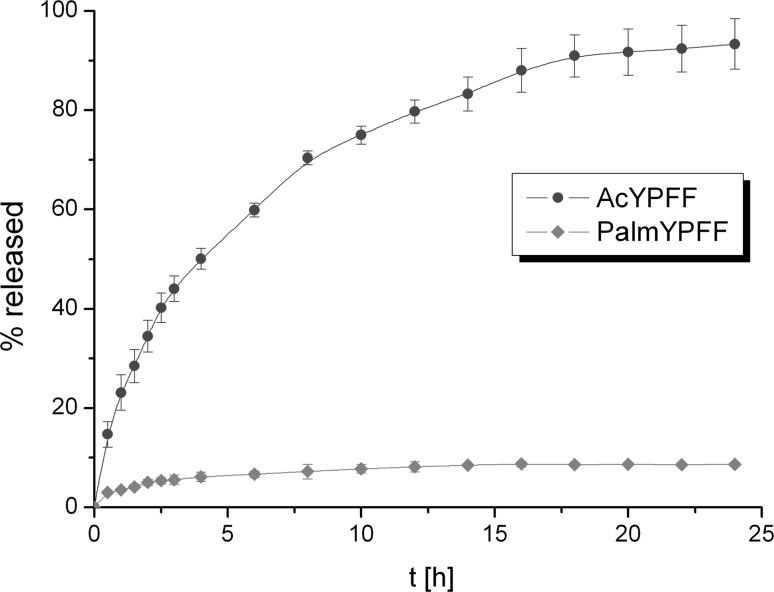
Comparison of the release profiles of AcYPFF and PalmYPFF from Hydrogels through Cuprophan membrane. Concentration of tetrapeptides in Hydrogels was 0.5%

The influence of the functional group on the release of tetrapeptide from Hydrogels was studied. This formulation was selected because the release efficiency of the active compound was the greatest from this semisolid. It should be mentioned that peptides are often modified to increase their stability and biocompatibility. In this study tetrapeptide YPFF was modified by introducing acetyl and palmitoyl groups. The release profiles of AcYPFF and PalmYPFF from Hydrogel are presented in Fig. 9. It was observed that the functional group influenced the release rate of the active compound. Much higher amount of active compound was released when tetrapeptide was functionalized with acetyl group compared to palmitoyl group. This could be explained by different solubilities of these tetrapeptide derivatives in both formulation and receptor fluid. The presence of long hydrocarbon chain in PalmYPFF influences its solubility in polar solvents; therefore, its release rate was much slower. It should be mentioned that the hydrogels obtained exhibit the same physicochemical properties although two different tetrapeptides were added. The difference in the release profiles is only related to the changes of functional group (Ac or Palm).

**Fig. 10:**
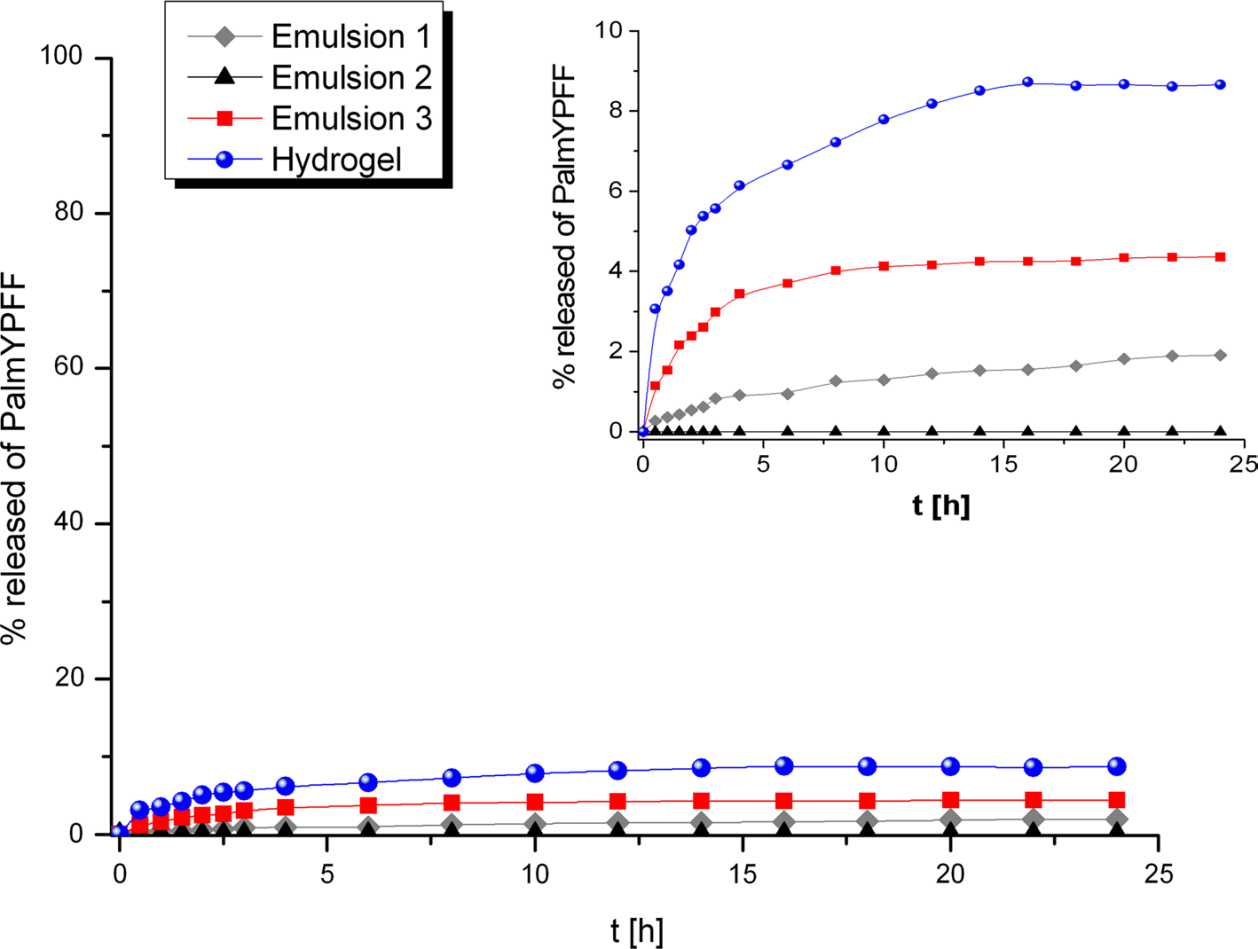
Release profiles of PalmYPFF from various formulations (Emulsions 1–3 and Hydrogel) through Cuprophan membrane

The release profiles of PalmYPFF from various formulations through Cuprophan membrane are presented (Fig. 10). The amount of PalmYPFF released from different formulations decreased in the following sequence: Hydrogel > Emulsion 3 > Emulsion 1 > Emulsion 2. Similar trend was observed when the AcYPFF was released from these semisolids. However, in the case of tetrapeptide modified with palmitoyl group, the amount of active compound that diffused through the membrane was much lower. Long hydrophobic chain changed the solubility of tetrapeptide both in the formulations and in receptor medium that had an influence on the slower release rate of PalmYPFF in comparison to release rate of AcYPFF. The highest amount of PalmYPFF was released from hydrogel that can be attributed to better availability of palmitoyl derivative in Hydrogel than in Emulsions. The release amount of PalmYPFF from Emulsion 2, which consists mostly of oil phase ingredients, was within error limit. Therefore, it could be suggested that the diffusion of PalmYPFF through oily phase might be a limiting step for its release.

## Conclusions

In this study semisolid formulations containing tetrapeptide YPFF were prepared and characterized. On the basis of the results obtained, it can be stated that the stability of emulsions with tetrapeptide is influenced by the preparation method and the presence of auto-emulsifier. The results proved that different factors had an impact on the release rate of active compound. The formulation type and its rheological properties strongly determined the permeation process of the tetrapeptide. The lower the viscosity of formulations, the faster is the diffusion of the active compound through the membrane. The kinetic data of Emulsions 1 and 2 showed good fit to the Higuchi model, whereas Emulsion 3 exhibit high linearity with Korsmeyer–Peppas model and Hydrogel with the first-order equation. Additionally, it was suggested that mechanism that led to the release of tetrapeptide was non-Fickian diffusion transport. It was also proved that nature of the membrane had influence on the release of tetrapeptide. Only hydrophilic membranes are appropriate for these studies. Furthermore, it was presented that much lower amount of tetrapeptide was released from formulations when it was modified with palmitoyl group compared to acetyl group. Long hydrophobic chain changed the solubility of the compound in both formulation and receptor fluid, and thus the release rate of the tetrapeptide was modified.
